# Microbiome-targeted dietary therapy in health and disease: Challenges, opportunities, and pathways forward

**DOI:** 10.1080/29933935.2026.2744027

**Published:** 2026-10-09

**Authors:** Alice S. Day, Anissa M. Armet, Srinivas Kamath, Paul M. Joyce, Robert V. Bryant, Heather K. Armstrong

**Affiliations:** a Department of Gastroenterology and Hepatology, Central Adelaide Local Health Network, The Queen Elizabeth Hospital, Adelaide, South Australia; b School of Medicine, Faculty of Health Sciences, Adelaide University, Adelaide, South Australia; c Basil Hetzel Research Institute, Adelaide, South Australia; d Department of Pediatrics, University of Alberta, Edmonton, Canada; e School of Pharmacy and Biomedical Science, Adelaide University, Adelaide, South Australia; f Department of Medicine, University of Alberta, Edmonton, Alberta, Canada; g Centre of Excellence for Gastrointestinal Inflammation and Immunity Research (CEGIIR), University of Alberta, Edmonton, Alberta, Canada

**Keywords:** Diet, gut microbiome, diet therapy gastrointestinal, disease

## Abstract

The gut microbiome has an established role in health and disease, and can be manipulated by diet, positioning microbiome-targeted dietary therapies as potential therapeutics. Mounting evidence supports dietary patterns rich in fermentable fibers and lower in nutrient-poor processed foods; however, there are individual, local, and system-wide complexities to adopting and sustaining dietary change as part of therapeutic dietary strategies. Wider adoption of diet therapy is also limited by critical factors that interfere and influence treatment outcomes, the most significant being inter-individual variability in response to dietary therapy, which remains poorly characterized and understood, hindering advancements in precision nutrition. This review examines the role of the gut microbiome in health and disease and describes barriers and solutions for developing and implementing microbiome-targeted dietary interventions as therapeutic strategies. We organize these barriers into three domains: biological, behavioral, and policy and systems, moving from molecular and individual-level mechanisms to population-level systems, and identify evidence gaps and candidate measurable outcomes to guide future research and dietary guideline development.

## Graphical abstract

**Figure:**
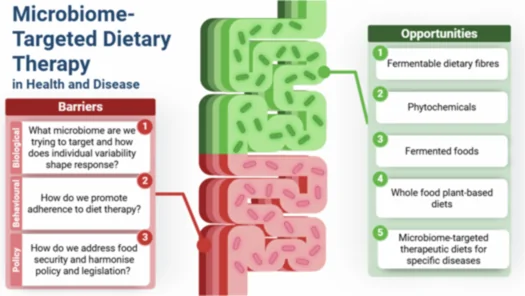

## 1. Introduction

Development and modulation of the gut microbiome is shaped by factors including birthing mode and exposures, medications, lifestyle (including diet), environment, genetics, and age.^1-4^ Once established, the diet continues to act as a primary fertilizer and manipulator of this complex ecosystem, with the ability to rapidly induce compositional and functional microbial changes within days.^5^ Epidemiological data correlate specific dietary patterns and consequent microbial composition and functional changes with chronic disease risk.^6-9^ Demand for microbiome-targeting therapies such as fecal microbiota transplant, probiotics, whole-diet strategies, prebiotics, and nutritional supplements is increasing, driven by consumer interest in therapies with a lower side effect and safety profile, a therapeutic ceiling for pharmacological therapies, and industry-driven economic potential.^10-12^ Diet is appealing, accessible, safe, and cost-effective, and holds promise as a microbiome-targeted therapy.^13^ However, prescription of and adherence to diet therapy remains challenging, limited by appropriate health, social, and economic infrastructure, and supporting population and industry-based legislation.^9^ ^,^ ^14^ Translatability of diet therapy between countries, even regions, is also an underexplored limitation, as evidence is lacking whether culturally-adapted dietary therapies are equally efficacious.^15^ ^,^ ^16^

Here, we explore how variations in the microbiome are linked with human health, along with describing barriers and solutions for developing and implementing microbiome-targeted dietary interventions as therapeutic strategies. We structured identified barriers into three domains, moving from micro- to macro-level: (1) biological barriers intrinsic to the individual and their microbiome, (2) behavioral barriers affecting dietary patterns, intake, and adherence, and (3) policy and systems barriers that shape the broader food environment. These domains are selected because each maps onto a distinct set of actionable levers – for clinicians and researchers, dietitians and patients, and policymakers, respectively – rather than as an exhaustive taxonomy of all possible barriers.

## 2. Role of the gut microbiome in health and disease

A stable and resilient gut microbiome is typically characterized by functional diversity and redundancy, symbiotically interacting with its host.^17^ ^,^ ^18^ Resident microbiota confer resistance to pathogen colonization by competing for nutrients and producing antimicrobial compounds, thereby suppressing pathogen expansion and limiting epithelial invasion,^19^ aiding in digestion, immune priming, and host metabolic regulation.^20^ A resilient microbiome is adaptive and able to sustain partial maintenance and often rapid recovery of key functions without significant disruption to host health following temporary disruption (e.g., after a short-term course of antibiotic therapy).^4^ ^,^ ^21^

An altered microbiome (dysbiosis), driven by diet and other lifestyle factors or host illness, diminishes colonization resistance, increasing susceptibility to enteric infection and promoting inflammatory feedback that can contribute to the burden of diseases.^17^ ^,^ ^22^ ^,^ ^23^ Long-term or permanent dysbiosis arises when this buffering capacity is lost, leading to altered community structure, functional redundancy, and a shift towards inflammatory and immunoregulatory perturbations, alongside altered neuroendocrine signaling and altered microbial signaling along the gut‒brain axis.^4^ ^,^ ^22^ ^,^ ^24^ However, causality remains debated, as microbial alterations may reflect both drivers and consequences of pathology.^25-29^

Microbial metabolism can activate, inactivate, or toxify dietary compounds, environmental chemicals, and pharmaceuticals before host absorption, altering bioavailability, efficacy, and toxicity, contributing substantially to inter-individual variability in the therapeutic response and treatment of disease.^30^ Several examples are described in the subsequent sections that illustrate that the gut microbiome is a potential mediator of interactions between diet and disease, offering opportunities to modify this interrelationship with targeted therapeutic strategies.

### 2.1. Opportunities for microbiome-targeted diet therapies

Evidence from human clinical trials demonstrates how various food components and dietary patterns benefit health via modulation of the gut microbiome (Figure 1). This knowledge can inform the application of microbiome-targeted diet therapies.

**Figure 1.**
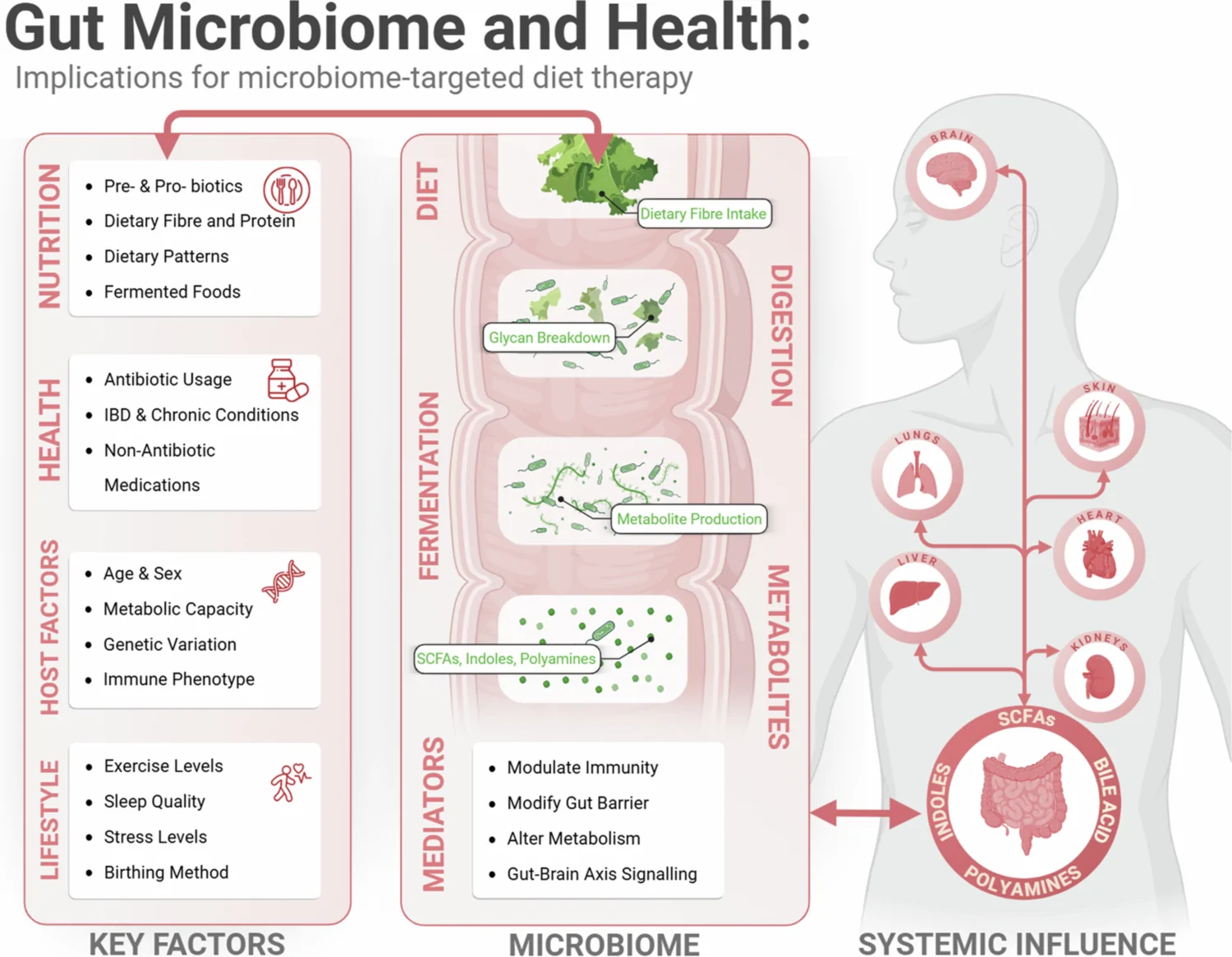
The gut microbiome structure and function as a mediator of diet-health interactions. Dietary inputs, host factors, and lifestyle exposures shape the gut microbiome composition and metabolic activity. Processed diets and medication exposures can disrupt microbial function, whereas fiber-rich and fermented foods promote microbial fermentation, generating bioactive metabolites, including short-chain fatty acids (SCFAs), indoles, and polyamines. These metabolites act locally to support gut barrier integrity, immune regulation, and colonization resistance and systemically to influence liver metabolism, cardiovascular risk, immune tone, and gut–brain axis signaling. Host-specific factors, including genetics, immune phenotype, and metabolic capacity, modulate these interactions, contributing to inter-individual variability in microbiome responses to diet and disease risk.

#### 2.1.1. Fermentable dietary fibers

Fermentable dietary fibers are carbohydrate polymers found in plant foods that cannot be broken down by endogenous enzymes in the small bowel, but are instead metabolized by gut microbes.^31^ Current knowledge is largely limited to how bacteria ferment fiber structures using microbial carbohydrate-active enzymes (CAZymes).^32^ Through cross-feeding, certain bacteria (primary degraders) initiate the breakdown of fiber glycosidic bonds, liberating saccharide compounds that can be further metabolized by other bacteria (secondary degraders).^33^ End-products of fiber fermentation, such as short-chain fatty acids (SCFAs), are then either utilized by other gut microbes or absorbed by the intestinal epithelium.^34^

Fermentable carbohydrates can elicit structure-specific changes to microbiome composition and function, as select microbial taxa with the enzymatic capacity to break down particular fiber structures often bloom in response to targeted supplementation.^35^ For example, supplementation of resistant starch type 4 (RS4) increased relative abundances of *Bifidobacterium adolescentis* and *Parabacteroides distasonis* across several intervention studies.^36-38^ Even small structural differences between two RS4s can direct increases in SCFAs propionate or butyrate due to different bacteria harboring distinct starch utilization pathways.^37^ This evidence has informed strategic supplementation of specific fiber structures to elicit predictable changes to the gut microbiome.^39^ ^,^ ^40^

Controlled intervention studies testing isolated fiber supplements, rather than fiber-rich whole foods or dietary patterns, allow for causal inferences on how dietary fiber affects health outcomes.^41^ Whether these health effects are then causally linked to fiber-induced changes to the gut microbiome remains challenging to study in humans,^42^ but integration of microbiome and clinical data from well-controlled trials provides insight. Supplementation of RS2 that was chemically modified to deliver high amounts of the SCFAs acetate and butyrate through microbial fermentation resulted in significant reductions in systolic blood pressure.^43^ Further, satietogenic effects of arabinoxylan supplementation were predicted by the bacterial taxa directly involved in its utilization.^44^ Both of these examples highlight the clinical relevance of harboring select microbial taxa capable of fermenting specific fiber structures in the treatment of metabolic diseases. Conversely, in established gastrointestinal disease, experimental *in vitro* data suggest that supplementing with individual fibers to induce compositional changes and restore microbial diversity may be insufficient to induce predictable compositional changes and improve disease outcomes.^45^ Thus, the targeted application of fermentable fibers should be informed by multiple factors (e.g., disease context, fiber structure) to optimize health outcomes.

**Table:** 

***Fermentable dietary fibers – gaps and limitations of current evidence*** *In vitro* and *ex vivo* mechanistic studies studying the effects of fermentable fibers on the gut microbiome have largely not been validated in randomized controlled trials, limiting their translatability. Placebo-controlled fiber intervention trials are difficult to conduct, as no truly inert placebo exists for powdered fiber supplements. Maltodextrin, a digestible carbohydrate, has been widely used, but it can induce microbiome changes and potentially induce inflammation.^46^ Microcrystalline cellulose, a non-fermentable fiber, has also been utilized as a control, but may have anti-inflammatory effects.^38^ ^,^ ^44^ Intervention studies are often conducted in healthy adults, with short durations (e.g., < 6 weeks) and small sample sizes. This particularly limits their applicability to disease states and therapeutic potential. Intervention studies have assessed a wide range of fiber types, physicochemical properties, and doses. The results are not broadly applicable to all fermentable fibers at various doses, and this fact may hinder the collation and interpretation of data for systematic reviews and meta-analyses. Causality is difficult to establish in humans; studies largely rely on associations between fiber-induced microbiome changes and health outcomes, which may inflate their clinical relevance.

#### 2.1.2. Phytochemicals

Phytochemicals are non-nutritive compounds that give plants their color, flavor, astringency, and smell.^47^ There are >26,000 phytochemicals in foods, which belong to different subgroups (e.g., flavonoids, glucosinolates, tannins).^48^ Phytochemicals vary in their bioavailability, but the majority (>90%) are unabsorbed in the small intestine.^47^ The gut microbiota possess extensive enzymatic capacity to transform phytochemical structures, which can greatly increase their bioavailability.^49^

Similar to fibers, phytochemicals are not only metabolized by gut microbiota but also directly affect microbiota composition and its functional output. Polyphenols can stimulate the growth of SCFA-producing microbes, such as *Faecalibacterium* and *Bifidobacterium* spp.,^50^ while flavonoids can enrich for the candidate probiotic species *Akkermansia muciniphila.* ^51^ Phytochemicals also act as antimicrobial agents, restricting the growth of pathogens and pathobionts owing to their ability to disrupt DNA synthesis and replication, and interfere with bacterial cellular structure and metabolism.^52^ Phytochemicals can further alter abundances of microbes involved in bile acid metabolism,^53^ as well as directly bind to bile acids to inhibit their re-absorption.^54^ Additionally, microbial tryptophan metabolism is affected through phytochemicals promoting taxa that produce bioactive indole derivatives (e.g., indole-3-acetic acid, indole-3-propionic acid) and kynurenic acid.^51^ ^,^ ^52^

Several clinical trials have reported significantly reduced circulating lipopolysaccharide levels, a marker of gut barrier function, as a result of polyphenol supplementation, which coincided with increased SCFA production.^55^ Further studies have also reported significant improvements to numerous cardiometabolic markers (e.g., inflammatory markers, blood pressure, blood lipids) that correlated with selective changes to gut microbiota.^56^ Thus, the gut microbiome can act as both a target and mediator of the benefits of dietary therapies involving high doses of phytochemicals.

**Table:** 

***Phytochemicals – gaps and limitations of current evidence*** Significant heterogeneity exists under the umbrella term ‘phytochemical’, with several sub-groups that differ greatly in structure and function. Health effects are structure-specific but are often not framed as such There remains a large ‘black box’ of undiscovered phytochemicals that are potentially relevant to health. Existing evidence for the mediating role of the gut microbiome in the health effects of phytochemicals is largely correlational. The prebiotic potential of phytochemicals remains to be causally validated. Intervention studies have been concentrated in adults with excess body weight, with limited data in gastrointestinal disease populations specifically.

#### 2.1.3. Fermented foods

Fermented foods are foods that have been modified by desirable microbial growth and enzymatic conversions of dietary compounds, often enhancing their nutritional profiles.^57^ Yogurt, miso, sauerkraut, kimchi, and natto have been included in traditional diets around the world for millennia, and are often included in national dietary guidelines.^58^

Non-heated fermented foods can provide high amounts of live microbes,^57^ including lactic acid bacteria, which can be detected in fecal samples, indicating that repeated consumption may promote persistence in the gut.^59^ Increasing the intake of fermented foods to ~6 daily servings increases microbiome diversity in healthy American adults.^60^ However, another longitudinal study in a rural Indian population associated long-term fermented food consumption with reduced diversity.^61^ Possible explanations for this discrepancy include differences in the specific fermented foods and microbial strains consumed, baseline microbiome diversity, and background dietary patterns between the two cohorts (e.g., fiber intake differences). This highlights that the impact of fermented foods on the microbiome may be context- and population-dependent. Numerous human trials have reported cardiometabolic benefits of fermented food consumption, particularly yogurt and kefir, though many have not directly tested effects on the gut microbiome in tandem.^62^ While the provision of live microbes and resulting changes to microbiome composition are potential mechanisms, there is currently insufficient evidence from human research to confirm this, necessitating additional well-controlled studies.^63^

**Table:** 

***Fermented foods – gaps and limitations of current evidence*** Very few controlled intervention trials have assessed high intakes of fermented foods; evidence is mainly derived from observational data. The effect of fermented foods on cardiometabolic outcomes is well established, but there is insufficient evidence for the impact of fermented foods in other clinical contexts (e.g., gastrointestinal diseases). Yogurt is the most studied fermented food, with limited experimental evidence for other traditional fermented foods (e.g., nattō, koko, kimchi, kombucha, etc.) To date, longitudinal studies have largely not assessed fermented food-induced changes to the gut microbiome, or have reported conflicting findings (e.g., effects on microbial diversity).

#### 2.1.4. Whole-food, plant-based diets

Whole-food, plant-based diets are either predominantly or entirely made up of vegetables, fruits, whole grains, legumes, nuts, and seeds, leading to high intakes of fermentable fibers, phytochemicals, and potentially fermented foods, with similar benefits as described above. Several iterations of these diets exist, allowing for flexibility in their targeted application for optimizing health. The Mediterranean diet (MedDiet) recommends high intakes of whole-plant foods, olive oil as the main fat source, and moderate (fish, poultry, and dairy) or limited (red and processed meats) intakes of animal products.^64^ In older adults consuming MedDiet for 12 months, levels of *Faecalibacterium prausnitzii* and *Roseburia* positively associated with improved cognitive function and negatively associated with inflammation and frailty.^65^ In women with impaired gut permeability, consuming MedDiet for 3 months improved markers of gut barrier function, mediated by diet-induced increases to propionate and butyrate.^66^ In adults with quiescent ulcerative colitis (UC), 12 weeks of MedDiet maintained clinical remission and was associated with increased production of SCFAs.^67^ Furthermore, adherence to MedDiet is also associated with improved cardiometabolic and mental health.^8^ ^,^ ^29^ Due to its strong evidence base, MedDiet is recommended for chronic disease prevention in several national dietary guidelines, as well as in certain clinical populations such as UC.^58^ ^,^ ^68^ This whole-food, plant-based diet can be modified to match cultural preferences while still maintaining its ability to modulate the microbiome and improve health.^69-71^

There is increasing recognition of how the modern lifestyle, including highly processed diets, adversely affects the gut microbiome and may predispose to immune-mediated chronic diseases and predict disease course.^72^ ^,^ ^73^ Healthy adults who consumed a non-industrialized-type diet based on rural Papua New Guinean dietary patterns [termed the Non-industrialized Microbiome restore (NiMe™) diet] experienced considerable cardiometabolic benefits after 3 weeks.^74^ The diet’s ability to significantly reduce cholesterol and inflammation levels was predicted by the restoration of microbiome features negatively impacted by industrialization, such as reduced abundance of pathobionts (e.g., *Bilophila*) and mucus-degrading CAZymes.^74^ In a randomized controlled trial involving healthy adult men, switching from a typical Western diet to a fiber-rich, heritage-style diet from the Kilimanjaro region for 2 weeks induced significant reductions in several inflammation markers, some of which were sustained after the intervention was completed.^75^ In a similar diet-switch trial, a high-fiber traditional African diet positively impacted colonic mucosal biomarkers of cancer risk, and was associated with lower mucosal proliferation, greater abundances of butyrate- synthesizing genes, and higher fecal butyrate concentrations.^76^ These interventions suggest that consuming non-industrialized-type diets can result in potent clinical benefits that are likely mediated by the restoration of positive diet-microbiome-host interactions.

**Table:** 

***Whole-food, plant-based diets – gaps and limitations of current evidence*** Not all whole-food, plant-based diets may elicit the same health benefits; effects are likely specific to certain properties (e.g., high unsaturated fat content of Mediterranean diets, very high fiber content of non-industrialized diets). Intervention trials have examined a wide range of outcomes (metabolic, immune, microbiome, etc.), which may limit data collation. It is unclear if the benefits observed are durable or cease after the intervention ends. Dose-responses (i.e., how much of the diet needs to be consumed) have also not been established. Intervention durations are short-term (e.g., 3 weeks, up to 1 y). Long-term randomized controlled trials are difficult to conduct but are necessary to establish how these diets influence hard clinical outcomes (e.g., development of chronic diseases) rather than only assessing surrogate risk markers. It is unknown whether findings from healthy adults generalize to other populations, including those with chronic diseases.

#### 2.1.5. Microbiome-targeted therapeutic diets for specific diseases

To date, the most successful examples of microbiome-targeted diet therapies have been used to induce and/or maintain remission in patients with IBD, particularly Crohn’s disease (CD). Exclusive enteral nutrition (EEN) is a fiber-free nutrition therapy widely used as a first-line therapy to induce remission in pediatric CD. Compared to steroids, EEN rapidly induces clinical response and early mucosal healing, though the mechanism of action is not fully elucidated.^77^ ^,^ ^78^ Microbial changes are observed in those who respond to EEN, with a reduction in diversity compared with healthy controls and those who did not respond to therapy.^79^ Furthermore, microbial signatures at baseline differed between responders and non-responders, consistent with other studies, suggesting a ‘microbial phenotype’ that is more responsive to EEN.^79^

There is increasing data supporting the use of food-based dietary induction therapies for CD. The CD exclusion diet (CDED) and Tasty & Healthy diet (T&H) have similar clinical efficacy to EEN in mild–moderate luminal CD.^80^ ^,^ ^81^ Both diets are designed to alter exposure to dietary components that modulate microbiome composition, gut barrier function, and inflammation; however, they differ in dietary prescription and, in contrast to EEN, encourage modest to high amounts of fiber.^80^ ^,^ ^81^ Positive clinical outcomes are often associated with CDED-induced shifts in microbiome composition,^82^ suggesting this microbiome-targeted dietary therapy was appropriately designed. Similarly, T&H increased SCFA-producers *Faecalibacterium prausnitzii* and *Bacteroides uniformis*, which suggests fermentable fibers had a therapeutic effect.^81^ ^,^ ^83^ However, when these three dietary strategies with a similar efficacy are compared, it remains unclear which specific dietary component(s) modulated the intraluminal environment favorably for therapeutic effect (i.e., changes in fiber, another dietary component, or nutrient interactions). Still, these findings collectively support the role of microbiome-targeted diets for CD treatment and provide promising insights that can inform the development of similar diet therapies for other immune-mediated diseases, especially those characterized by microbiome perturbations.

**Table:** 

***Microbiome-targeted therapeutic diets – gaps and limitations of current evidence*** Dietary therapies have largely been developed and validated for patients with IBD, particularly CD, with most intervention studies being conducted in pediatric populations. Translation into adults with IBD, as well as other clinical populations, is not well understood. Intervention studies in CD often compare a new diet-based therapy in isolation to EEN. There is limited experimental evidence for the effectiveness of these dietary therapies as treatment adjuncts (i.e., provided alongside biologics or advanced therapies). Uptake of diet-based therapies may be limited when established pharmacological therapies predominate in clinical practice, which can hinder research on diet as a therapeutic treatment or adjunct. Currently unable to isolate which dietary component(s) of the therapeutic diets drives their benefits, which may limit effective modifications to diet protocols for cultural, religious, or other reasons.

## 3. Barriers and determinants of successful microbiome-targeted dietary interventions

While compelling evidence for microbiome-targeted dietary therapies to manage health and disease exists, there are pragmatic challenges to consider beyond the dietary therapy itself (Figure 2).^84^ These challenges can be organized into three domains, moving from micro- to macro-level: (1) biological barriers at the level of the individual and their microbiome, (2) behavioral barriers affecting uptake and adherence, and (3) policy and systems barriers that shape the food environment. These domains are illustrative rather than exhaustive, selected as the categories most directly actionable by clinicians, researchers, and policymakers, respectively.

**Figure 2.**
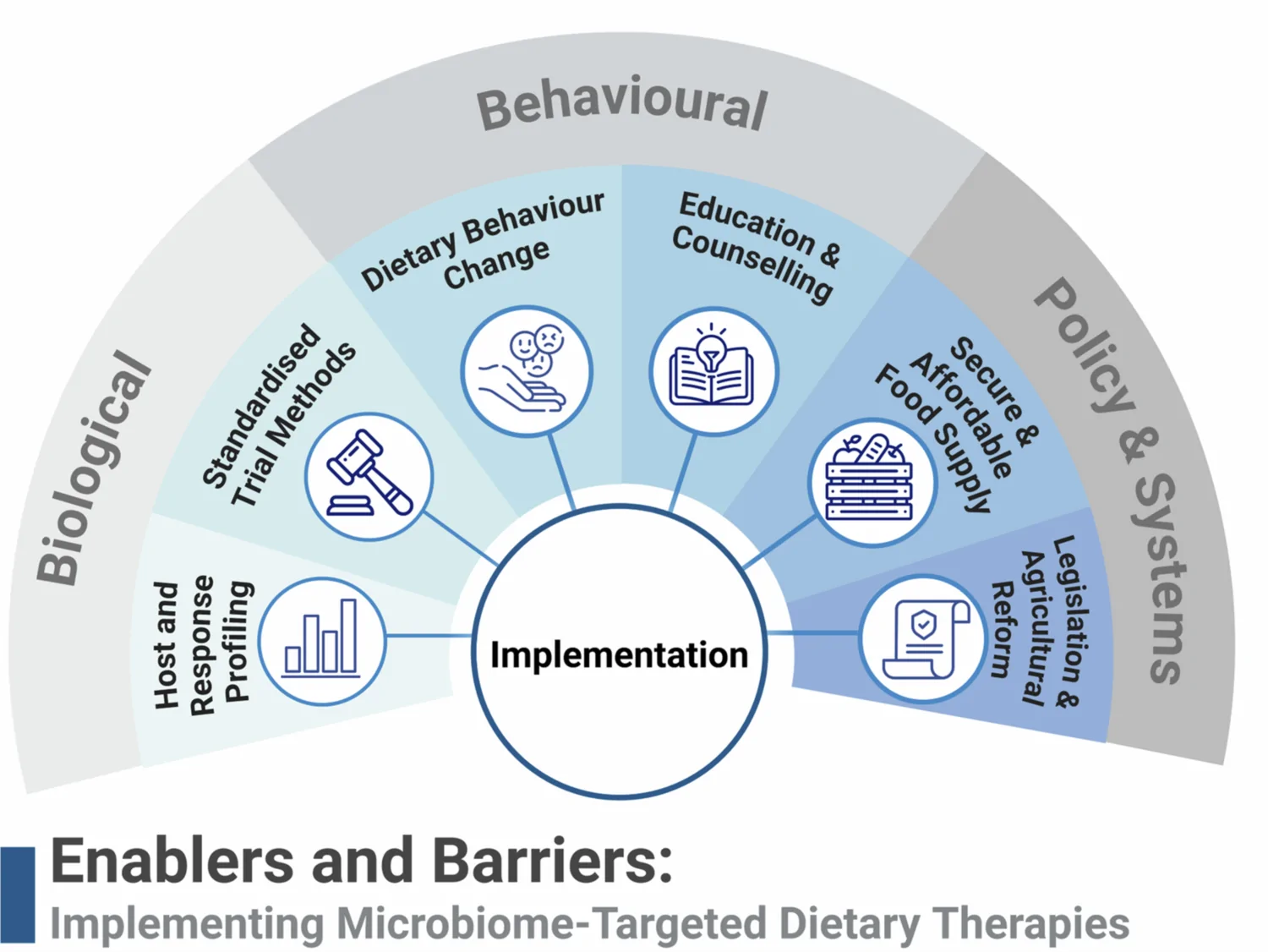
Enablers and barriers to implementing microbiome-targeted dietary therapies.

### 3.1. Biological barrier: what microbiome are we trying to target, and how does individual variability shape response?

The overarching goal of microbiome-targeted dietary therapies has been to induce putatively beneficial changes, such as increasing fiber-fermenting bacterial species (e.g., *Bifidobacterium, Faecalibacterium*) and fungi, while decreasing pathobionts (e.g., *Bilophila wadsworthia,* certain Proteobacteria) and ethanol-producing microbes.^85-87^ However, while some individuals possess a readily modifiable gut microbiome, others are more resistant to change.^88-93^ Microbiome-targeted dietary interventions can induce high inter-individual variability in responses, potentially owing to differences in baseline nutritional status, circadian biology, environmental exposures, geography, pharmaceutical exposures, inflammation, and various social determinants of health.^94^ These factors need to be understood for their role in the host response to diet before microbiome-targeted interventions can be broadly implemented. Large, adequately powered clinical cohorts capable of accounting for genetics, sex, age, ethnicity, medical history, social and environmental factors are required. Furthermore, standardizing data capture on habitual diet and methods to sequence and analyze metagenomics data are also critical.^94^

#### 3.1.1. Individual and physiological determinants of microbiome response

Individual response to therapy relates to a change in relevant metrics of health. For example, a change in inflammatory markers in someone with IBD after commencing therapy compared with someone who does not respond.^80^ ^,^ ^95^ ^,^ ^96^ Significant inter-individual microbiome variability can influence responses to microbiome-targeted therapies.^79^ Individual determinants of microbiome responses include intestinal pH, metabolic cross-feeding of nutrients, and production of gaseous and other microbial-derived metabolites. Environmental factors beyond diet, such as medications, are another potential determinant. Two examples of commonly prescribed medications illustrate this point – proton-pump inhibitors (PPI) and 5-aminosalicylic acid (5-ASA) compounds. Approximately 1 in 4 adults globally are prescribed PPIs,^97^ which suppress stomach acid production, affect intestinal pH, and are associated with reduced microbial diversity and compositional changes compared to healthy controls.^98^ Downstream effects of PPI use include alterations to the digestion and fermentation of fiber and protein, which have implications for microbiome-targeted dietary interventions and microbial degradation, and the activation of drugs.^99^ The second example, 5-ASA compounds, are a first-line anti-inflammatory therapy for ulcerative colitis (UC), an inflammatory bowel disease (IBD), that relies on pH-dependent release systems to deliver the active drug to the colon. Altering the consumption of dietary fiber can inhibit or enhance the luminal release of these compounds, mediated through the physiological effects fiber has on gut transit time, microbial-derived fermentation of fibers, short-chain fatty acid (SCFA) production, and associated changes in luminal pH.^100^ Interestingly, in a Norwegian cohort of 10,149 adults with newly diagnosed UC, PPI use was an independent predictor of escalation to advanced therapies and a more severe disease course, suggesting potential broader downstream drug-microbiome-diet interactions remain poorly understood.^101^

Distinct microbial signatures (altered diversity) are observed in health and disease.^79^ ^,^ ^102^ ^,^ ^103^ This has important implications for microbial function, metabolic cross-talk and overall health of the microbial ecosystem, whereby differences in the patterns of fermentation, production and uptake of gaseous metabolites may have therapeutic or pathogenic implications.^104^ For example, in cultured intestinal biopsies of participants with and without IBD, unfermented dietary *β*-fructans induced the production of pro-inflammatory cytokines in patients with active IBD, but not patients in remission or those without IBD, results that were confirmed in a clinical trial that showed select patients had a pro-inflammatory response to *β*-fructan supplementation.^105^ The extent to which these community variations affect response to dietary interventions remains an evolving area of science that needs to be better understood in larger-scale intervention trials.^12^ ^,^ ^79^ ^,^ ^106^

### 3.2. Behavioral barrier: how do we promote adherence to a dietary intervention?

#### 3.2.1. Adherence to diet therapies

Adherence is one broad determinant of a successful prescribed dietary intervention that relates to how dietary behavior aligns with the implementation of dietary advice.^107^ Family, cultural, and food-based traditions, socioeconomic status, health literacy, cooking skills, lifestyle constraints, and co-existing health conditions treated with diet may affect an individual’s ability to adhere to a dietary intervention.^108-110^ For example, a diet that emphasizes home cooking and avoiding processed convenience foods may be challenging for someone who lacks cooking skills and basic food knowledge, or access to cooking tools.^111^ ^,^ ^112^ In the context of therapeutic diets for chronic disease management, frequent access to evidence-based, specialist dietary counseling and support is associated with greater adherence to dietary therapy and treatment response.^113^ ^,^ ^114^ Factors that influence dietary adherence would indirectly result in inadequate microbiome modulation, potentially limiting the health benefits of the dietary strategy.

Longitudinal cohort studies suggest adherence to population-based healthy eating guidance is poor, despite public health education efforts.^9^ ^,^ ^115-118^ Social studies suggest being diagnosed with a health condition is a stronger motivator for committing to dietary changes compared to eating well as a preventative health strategy.^115^ ^,^ ^119^ Harnessing cohort or population-specific motivational enablers to achieve long-term dietary change is imperative for prescribers of microbiome-targeted dietary interventions, best addressed through nutritional assessment and evidence-based dietary counseling.^120^ Failure to do so may affect adherence, limit favorable microbial changes, and subsequent therapeutic response. Dietary counseling is, however, an individual-level intervention that is difficult to scale to population-wide adoption unless supported by system-level change, such as improving access to dietitians and incorporating medical nutrition therapy into insurance and reimbursement structures; this distinction is addressed in the following section.

### 3.3. Policy and systems barriers: how do we address food security and harmonize policy and legislation?

#### 3.3.1. Food security, source, and supply

Since microbiome-targeted dietary therapies rely on the sustained intake of specific whole foods or dietary components (e.g., fiber structures, phytochemicals), they are especially sensitive to barriers related to food source, supply, and security, more so than pharmacological therapies that do not depend on food access.

A high-quality, affordable, and accessible food supply influences the implementation of population-based dietary guidance and microbiome-targeted dietary interventions. The most widely researched dietary pattern, MedDiet, consistently demonstrates population-based benefits for chronic disease^121-124^; however, pragmatic translatability of this dietary pattern in non-Mediterranean countries while achieving the same health outcomes is not necessarily feasible or known to be therapeutically equivalent.^71^ ^,^ ^125^ Future intervention trials need to assess culturally-relevant iterations of the Mediterranean diet alongside longitudinal microbiome and clinical outcomes to help inform future population-specific dietary policies.

Geographical availability and access to fresh fruit and vegetables is an important community-wide determinant of diet quality and a diverse microbiome.^126^ With urbanization and time-related longitudinal cultural changes, access-affordability and widened social inequalities are observed globally. Households have less opportunity to produce food, and access to local food markets is variable and can be more expensive.^127^ Farmers are increasingly facing environmental and financial challenges to maintain viable agricultural practices, and regional communities face food supply-chain barriers to accessing fresh produce.^127-129^ Affordability of a secure food supply influences dietary patterns and creates inequalities in lower socioeconomic communities to follow healthful diets.^130^ For example, in the rural United States, increased availability of cheap, calorie-dense foods in local shops is identified as a key community barrier to eating fresh fruit and vegetables,^131^ ^,^ ^132^ and a poor-quality diet, low in fresh fruits and vegetables, wholegrains, nuts and seeds, is associated with a ‘starved’ microbiome which lacks diversity and is associated with chronic disease risk.^87^ ^,^ ^126^

Food source, supply, and security are critical barriers to adhering to whole-food diets like the MedDiet. Action is required at local council and government levels regarding community infrastructure (e.g., supermarkets, fresh produce stores, density of fast food chains), town planning (e.g., footpaths, cycling routes, public transport, urban farms) and food supply-chain networks (e.g., transport and flow of fresh produce to urban, regional, and remote areas) to support a community’s ability to adhere to healthful dietary patterns that support the gut microbiome, along with versatile public health dietary education that meets the needs of the population to help mitigate diet-related chronic disease burden.^133^ ^,^ ^134^

#### 3.3.2. Food systems and agriculture prioritize profit, quantity, and shelf-stability over nutritional quality

Here, we use the term ‘processed foods’ in reference to food-processing classification frameworks such as NOVA, which group foods by the extent and purpose of industrial processing rather than by nutrient content alone.^135^ On a global scale, environmental and agricultural factors (e.g., geographic availability, season, and climate) as well as industrial factors (e.g., chain supply, food processing techniques to improve product stabilization, palatability, and shelf-life) influence food supply and diet quality.^136^ Nutritional food quality may be compromised by food processing factors.^136^ ^,^ ^137^ Emerging evidence for the chronic health impacts of how industrialization has influenced food supply suggests this may be responsible for the loss of commensal microbes.^138^ Differences in phylogenetic diversity and functional capacity of the gut microbiome observed in hunter-gatherer tribes in Tanzania compared to Europeans with a Westernized diet suggest all these factors have had a critical influence on indigenous gut microbiota,^139^ and further support the need for microbiome-targeted interventions appropriate for indigenous populations.

Nutritional and food policies that result in barriers to high-quality nutrition and application of microbiome-targeted diet therapies in the field of medicine often stem from structural, financial, and regulatory gaps rather than direct prohibitions.^140-145^ Factors influencing access can include restrictive insurance coverage, as food is not typically recognized as a medical intervention outside of select medically-tailored meals and supplements. An example of this is regulation around EEN – even with strong evidence as a cost-effective, efficacious microbial-targeted induction therapy for CD, access to EEN is limited.^146^ Such funding for diet therapy is typically limited to acute care or specific, narrow circumstances. Beyond defined clinical indications for diet therapy, population-based dietary recommendations are administered through national food-based dietary guidelines as a preventative strategy for chronic disease,^147^ and adherence is left to the individual, with a general failure to embed nutritional screening, counseling, and referrals directly into medical care in clinical settings so that individuals can understand their diet and disease risk.^148^ Meanwhile, many tertiary clinical settings lack the infrastructure to store fresh produce or the staff, time, and resources to maintain and deliver high-quality, fresh food diets, acting as another barrier to providing patients with first-line whole-food diet therapy as part of a broader strategy to educate on nutrition as medicine.^149^ Nutritional policies primarily focus on negative feedback regarding nutrition in health, such as warning labels and taxation systems, rather than on positive strategies to increase the intake of healthful foods to support whole body health, including a diverse, resilient microbiome. Such policies also often fail to address food inequity and social determinants of health, such as lack of a living wage or limited access to healthy food, potentially widening, not bridging, towards health equality.^130^ ^,^ ^150^

Industrial and agricultural food policies often prioritize high-yield, low-cost commodity production over nutritional quality, acting as systemic barriers to accessing fresh and nutrient-dense food that supports a highly functioning microbial ecosystem.^150-152^ These policies foster a food environment where calorie-dense, nutrient-poor, highly-processed foods are more accessible and affordable than healthy, whole foods.^150-154^ Certain agricultural policies prioritize subsidies for commodity crops, including corn, soy, and wheat, which are staple foods primarily used for processed food ingredients, livestock feed, and biofuels, rather than direct human consumption of fresh fruits and vegetables. Agricultural policies often focus on production volume, yield, and income rather than dietary quality, leading to a disconnect between what is grown and what is needed for public health.^150^ ^,^ ^152^

Agribusiness lobbying can shape national dietary guidelines, prioritizing industry profits over public health; for example, promoting consumption of meat and dairy while resisting evidence-based recommendations for plant-rich diets to support a diverse, healthful microbiome and a lower risk of chronic disease burden.^126^ ^,^ ^150^ Industrial agriculture can prioritize input-intensive, large-scale monocropping, which can result in soil nutrient depletion and loss of nutrient contents that are further depleted by food processing and storage practices.^152^ Policies that fail to restrict the marketing of low-nutritional-value foods and allow misleading health claims on labels exacerbate poor dietary habits and risk compromising microbial diversity.^152^ Furthermore, limited investment in the processing, storage, and distribution of perishable produce creates logistical “food desert” barriers that make high-quality fresh foods less available compared to packaged goods.^152^ These policies and procedures can result in reduced nutritional value of crops, with studies showing significant declines in calcium, iron, and vitamin C levels in fruits and vegetables over the past 60 y.^152^ In conjunction, epidemiological studies have associated calorie-dense, highly processed foods with epidemics of obesity, diabetes, and other chronic diseases,^17^ ^,^ ^155^ whereas native, locally-sourced, non-industrialized dietary interventions have shown potential to reverse these cardiometabolic effects.^74^ Ultimately, shifts in diet quality and increases in chronic health issues have been linked with compositional and functional changes in the gut microbiome (e.g., phylum-level changes in abundance, reduced diversity, altered bacterial gene expression resulting in changes in metabolic function with obesity).^58^ ^,^ ^156-158^

To counter these factors, global health experts continue to provide evidence in support of diverse, nutrient-dense whole foods to promote improved health outcomes.^40^ Public health programs such as the North Karelia project in Finland are broadening collaborations to engage system-wide change across health services, schools, non-governmental organizations, food industry, and media to influence chronic disease burden.^159^ Advancements in agriscience research are also being observed. For example, wheat is a global dietary staple with widely recognized cardiometabolic health benefits in its unrefined, nutrient-dense wholegrain form.^40^ ^,^ ^160^ ^,^ ^161^ Milled white-wheat flour remains one of its most widely consumed forms in the Western diet, lacking the health benefits of whole-grain wheat. Research into genetic improvements and enrichment of wheat starches (e.g., amylose and resistance starch) and fermentable fibers (e.g., *β*-glucan and arabinoxylan), along with changes to wheat milling and processing techniques, anticipates marked improvement in the nutrient density of wheat flour as a step toward improvement in diet quality, which can then be evaluated for microbial changes and health outcomes.^40^ ^,^ ^161^

## 4. Opportunities to advance the development and use of microbiome-targeted diet therapies

While data continues to emerge regarding the gut microbiome’s role in regulating host response to nutrition and our capacity to provide optimal nutrition care, there is a mounting appreciation for the complexities of food, the potential of dietary therapy, and the individualized responses to dietary change.^162^ ^,^ ^163^ The resulting health outcomes associated with consuming different diets vary significantly among people based on the biological, behavioral, policy, and legislative factors discussed, and these must be taken into account when interpreting data and designing future studies.^94^

### 4.1. Research gaps in developing microbiome-targeted dietary therapies

While research continues to define specific microbiota and functions linked to health, there is a global need to better understand how habitual and traditional ‘native’ diets can be improved and modified to support a healthy microbiome. Evidence suggests restrictive diets are not effective for everyone, and short-term diets do not necessarily induce long-term favorable microbial changes,^164^ while dietary supplements such as fermentable fibers can contain contaminants (e.g., chemicals and microplastics) and lack important phenolics or other nutrients.^165-167^ Furthermore, the supplement extraction process can require solvent exposure to purify the specific molecules from their natural food sources. Whole-food sources are considered superior not only because of their nutrient composition, but also because they may have broad impacts on the gut microbiota rather than on selective microbes impacted by single fibers and other supplement food molecules.^165-168^ Combined approaches, such as dietary interventions with live-microbe biotherapeutics (e.g., FMT), may offer further improvement of gut microbiome composition and functions, and warrant further investigation.^169-174^

Mounting evidence extends our existing understanding beyond the limitations of ‘one-size-fits-all’ strategies toward an individualized approach to modulation of the gut microbiome for therapeutic effect.^175^ Precision nutrition is a future direction for personalized diet therapies, using microbial and metabolomic biomarkers along with baseline health status, body composition, and cultural preferences unique to an individual to guide and titrate the therapeutic application of diet, appreciating that dietary therapies may effectively modulate different aspects of the microbiome in an individualized manner.^162^ However, while initial reports are both promising and hypothesis-generating, precision nutrition is still in its infancy and requires extensive validation before it can be implemented at-scale as a treatment strategy.

### 4.2. Dietary guidelines need to be inclusive of microbiome science

While population-based dietary guidelines provide a dietary scaffold for eating well, many are outdated, lag behind science, and largely do not make reference to microbiome science.^58^ ^,^ ^176^ For example, the Australian Dietary Guidelines focus on nutrient intake and adequacy to prevent chronic disease rather than optimizing microbial composition and function to reduce chronic disease risk.^147^ Although consensus is still lacking on what constitutes a healthy microbiome, the existing nutritional microbiology evidence base can already inform specific microbiome outcomes for dietary guidelines to target to elicit positive health outcomes. For example, reduced colonic pH signals high saccharolytic fermentation and SCFA production, and is supported by high fiber intake.^177^

Validation of candidate, measurable microbiome outcomes is required to inform future guideline development, especially distinguishing surrogate compositional markers from functional and clinical endpoints. Compositional markers, such as the relative abundance of implicated taxa (e.g., *Bifidobacterium, Faecalibacterium prausnitzii, Akkermansia muciniphila* as favorable markers, and *Bilophila wadsworthia* as a pathobiont marker), are the most accessible to measure but have not been consistently validated against health outcomes. Functional and metabolomic markers, including SCFA production (particularly propionate and butyrate), circulating lipopolysaccharide as a marker of gut barrier integrity, bile acid profiles, and tryptophan-derived indoles, sit closer to the causal pathway between diet, microbiome, and host physiology, and are supported by the intervention evidence discussed earlier. However, an international consensus statement on microbiome testing in clinical practice recently concluded that evidence supporting the clinical usefulness of compositional microbiome testing remains insufficient, and called for large-scale trials that measure hard clinical endpoints (e.g., disease remission, cardiometabolic outcomes) rather than surrogate biomarkers alone before microbiome-informed metrics are incorporated into dietary guidance.^178^ Prioritizing this hierarchy of outcomes, from compositional signals to functional and metabolomic evidence to hard clinical endpoints, would help ensure that future incorporation of microbiome science into dietary guidelines rests on outcomes proven to matter for health, rather than on measurement convenience alone.

### 4.3. Integration of microbiome testing and manipulation into healthcare

There remains a lack of actionable recommendations to implement integrated nutrition care.^96^ ^,^ ^105^ ^,^ ^179-182^ Preliminary studies demonstrated microbial functions, such as the ability to ferment dietary fibers or produce microbiota-derived bioactive metabolites, can be used to define precision nutrition relevant for different health settings.^96^ ^,^ ^105^ ^,^ ^179-182^ As novel disease-specific biomarkers are discovered, modern tools such as wearable devices are able to provide live-monitoring, while machine learning models can be leveraged to deliver personalized diet and lifestyle recommendations. While automated tools can support routine assessments, meal tracking, and education, delivering such precision nutrition approaches requires high-level clinical decision-making that necessitates clinical support from dietitians.^183-187^ These technologies also raise technical and ethical considerations that warrant further attention, including the accuracy and validation of consumer-grade sensors and algorithms against clinical-grade measures, data privacy and security for sensitive microbiome and health data, equity of access, and the risk of algorithmic bias where training datasets under-represent certain populations.

## 5. Conclusion

Microbiome-targeted dietary interventions show great potential as therapeutic agents to modulate disease, but their translation into standard practice remains limited by challenges spanning biological, behavioral, and policy and systems domains, moving from the micro-individual level through the macro-level of food environment and policies that shape population health. Combination approaches and precision nutrition hold promise as the next frontiers in microbiome-targeted diet therapies to overcome inter-individual variability in responses, provided that this promise is matched by trials that prioritize standardized, clinically meaningful microbiome outcomes over compositional signal alone. An integrated approach must be adopted across these three domains, alongside advancements in, and harmonization of, microbiome science to accurately evaluate treatment efficacy and outcomes at both individual- and population-based levels.

## Data Availability

N/A. No supporting data to declare.

## Social media promotion

Diet fuels the gut microbiome – but not everyone responds the same. This review unpacks biological, behavioral, and system-wide barriers and solutions to advance microbiome-targeted diet therapies for health and disease.
